# Motivations and Barriers Associated With Physician Volunteerism for an International Telemedicine Organization

**DOI:** 10.3389/fpubh.2019.00224

**Published:** 2019-08-13

**Authors:** Erin J. Kim, Steven Fox, Meghan E. Moretti, Michelle Turner, Timothy D. Girard, Stephen Y. Chan

**Affiliations:** ^1^The Addis Clinic, Inc., Nashville, TN, United States; ^2^Department of Medicine, University of Pittsburgh Medical Center, Pittsburgh, PA, United States; ^3^Department of Critical Care Medicine, Clinical Research, Investigation, and Systems Modeling of Acute Illness (CRISMA) Center, University of Pittsburgh School of Medicine, Pittsburgh, PA, United States

**Keywords:** telemedicine, global health, physician volunteers, international volunteerism, frontline health workers, motivations, barriers, humanitarian

## Abstract

**Introduction:** The Addis Clinic uses volunteer physicians to implement an international humanitarian telemedicine program. We sought to identify motivations and barriers that may contribute to physician volunteerism in international telemedicine.

**Methods:** We surveyed active and inactive volunteers working with The Addis Clinic. Descriptive statistics were used to examine closed-ended questions, while a qualitative approach identified overarching themes for open-ended questions. The Volunteer Functions Inventory framework was also applied.

**Results:** Among 69 active and 25 inactive volunteers, survey response rates of 74 and 72%, respectively, were attained. Volunteer cohorts exhibited comparable distributions across sex, marital status, and children. Active, as compared with inactive, participants were significantly more likely to be <40 years old (51 vs. 39%, *p* = 0.01), have prior experience with international/global health (67 vs. 39%, *p* = 0.04), and express an interest in international/global health work (82 vs. 50%, *p* = 0.008). Active volunteers were predominantly concerned with challenges regarding patient care: they more often reported the asynchronous nature of communication with frontline health workers as a significant barrier (37 vs. 6%, *p* = 0.047), and increased patient follow-up significantly drove their enthusiasm (64 vs. 35%, p = 0.05). Conversely, active volunteers were less likely to cite commitment/availability as a significant barrier for participation (33 vs. 72%, *p* = 0.002), less likely to be incentivized by opportunities to fulfill professional obligations (14 vs. 59%, *p* = 0.001), and more likely to be satisfied with the telemedicine experience (86 vs. 0%, *p* < 0.0001). Opportunities to receive remuneration or recognition did not increase the likelihood of volunteering for either cohort. Malpractice concerns were cited in a comparable minority across cohorts (20 vs. 17%).

**Conclusions:** Age and global health experience/interest were significant predictors of physician volunteerism. While inactive volunteers reported time commitment as a barrier, active participants were concerned with challenges regarding patient care and motivated by increased methods to connect with patients. Financial considerations and recognition were infrequently reported as a barrier. With advances in telemedicine globally, results from this study can be used by organizations involved in international telemedicine to develop effective volunteer recruitment and retention strategies.

## Introduction

Many remote, hard-to-reach communities in low- and middle-income countries (LMICs) suffer from a shortage of skilled health workers (1). These communities struggle with retaining and attracting a qualified health workforce, and at times, are served by a single frontline health worker (FHW) with limited clinical training (2). Patients with complex conditions and other chronic illnesses may need to travel long distances across difficult terrain to receive specialized medical care (3). Many of the FHWs in these communities are isolated and lack the type of professional support found in urban areas. Addressing workforce challenges has the potential to improve patient outcomes and build stronger health systems (4). Consequently, demand for volunteers with medical expertise has risen significantly as communities and governments lack the financial resources to recruit, train, and maintain local health workers (5, 6).

Many of the volunteers serving in these communities are healthcare professionals from developed countries seeking global health experiences for humanitarian and/or career-related reasons. Volunteer assignments are typically short-term placements lasting anywhere between 2 and 3 weeks to <2 months. This form of volunteerism, while popular, raises important ethical considerations. For instance, local organizations that host international volunteers have expressed concerns about temporary volunteers competing with, or even substituting, locally trained health workers (5, 7). Additionally, the sustainability and effect of using short-term volunteers to supplement the local health workforce have been questioned (6).

With the advances in mobile technology and the number of internet users on the rise globally, there is an emerging consensus among key stakeholders that ICT4D (Information and Communications Technologies for Development) interventions have the potential to strengthen FHWs' clinical knowledge and improve patient outcomes (8–10). Organizations supporting FHWs in rural communities have used technology to train, motivate, and share current medical guidelines, as well as create a virtual community of health workers (11, 12).

Telemedicine technology is one example of an ICT4D intervention that can be used to support local health systems by giving FHWs continuous access to medical experts located remotely. Evidence suggests that digital telemedicine tools have the potential to strengthen health systems by supporting and training FHWs in geographically separate communities (8, 13). Additionally, international telemedicine organizations provide timely access to specialists, reducing the need for patients to travel long distances for specialized medical care (14–16). Brandling-Bennett et al., found that consultations via e-mail between physicians in Boston, Massachusetts with a mobile nurse in Cambodia reduced the number of patients requiring outside referrals (17).

The Addis Clinic is one such nonprofit organization that uses telemedicine to address many of the concerns highlighted by local organizations in LMICs. The Addis Clinic connects FHWs working in low resource areas with a network of specialty physician volunteers, offering a mechanism for communication of medical information and recommendations through its internet-based platform^1^. Namely, The Addis Clinic facilitates the exchange of medical knowledge through teleconsultations submitted by FHWs that are then triaged to the appropriate physician volunteers. Based on a “store-and-forward” technology, this platform is ideal in low resource settings with limited connectivity^2^. It allows FHWs to initiate and store teleconsultations on their devices without internet or satellite connectivity. As soon as FHWs are within range, they can submit their saved cases to physician volunteers located in geographically separate areas of the world.

While international telemedicine organizations such as The Addis Clinic have the potential to strengthen the health workforce in remote, resource constrained communities, they rely heavily on physician volunteerism (16, 18). In many ways, telemedicine can facilitate participation of physicians who otherwise would not volunteer to serve internationally. Namely, it eliminates the barrier of traveling abroad for physician volunteers and allows them to volunteer from their location of choice. This encourages long-term volunteerism and can offer sustainable support for communities in resource constrained areas. Furthermore, it addresses the challenge of partner organizations being susceptible to gaps in the provision of health services with short-term volunteers.

Yet, the motivations and barriers of international volunteers in organizations such as The Addis Clinic are not obvious. Researchers examining healthcare professionals returning from short-term medical trips have found that most international volunteers are motivated for altruistic reasons, but self-interested motives such as the opportunity to travel also played a role (19). Furthermore, prior researchers have assessed the effect of international volunteers on local health systems, as well as perspectives from partner organizations (5, 6). While many researchers have evaluated the various experiences of international volunteerism, few have examined the motivations and barriers of healthcare professionals volunteering virtually. In this study, we sought to identify motivations and barriers that may contribute to physician volunteerism in international telemedicine operations, thus providing quantifiable data to develop effective recruitment and retention strategies in the future.

## Materials and Methods

### Survey Instrument and Participants

An initial email invitation with a personal link to the survey was sent to active (69) and inactive (25) physician volunteers for The Addis Clinic in November of 2018. Survey responses were collected for 1 month with targeted follow-up emails sent 2 and 3 weeks after the initial invitation. Reminders about the survey were included in the physician newsletter and social media. Active volunteers were defined as physicians currently on the roster for teleconsultations and have consulted on a case within the past 12 months. Inactive volunteers were defined as physicians who have expressed interest in volunteering with The Addis Clinic, but never onboarded or have not consulted on a case within the past 12 months. More than 70% of the physician volunteers started volunteering in 2017, while fewer than 10% had been involved with the organization since its inception in 2011. The remaining 20% of volunteers onboarded between 2012 and 2017. Volunteer turnover has been low in recent years with nearly 90% of volunteers leaving the organization before 2014.

The University of Pittsburgh online survey system (Qualtrics) was used to distribute the survey. The survey consisted of 30 close-ended (e.g., multiple choice, Likert-type scale, dichotomous) and four open-ended questions. Questions were designed to assess volunteers' level of satisfaction with and perceptions regarding motivations and barriers to working with The Addis Clinic. The survey also included a demographic section to better understand background characteristics of the respondents. Survey results were organized into Demographics, Volunteer Experience/Background, Barriers to Volunteer Work, and Motivations to Volunteer Work. Findings under Volunteer Experience/Background include survey questions designed to assess participants' experience with global health and telemedicine, in general. The questions were developed in collaboration with several Addis Clinic staff members, to include points that have been identified as potential factors influencing volunteers' perceptions of their experience with The Addis Clinic and their motivations to volunteer. Prior to dissemination, the survey was piloted with two physician volunteers, as well as reviewed by a survey expert to improve the accuracy of the instrument. The survey was approved by the University of Pittsburgh Medical Center (UPMC) Institutional Review Board (PRO18060522). All participants were given the opportunity to opt-out of the survey. Participants were not required to complete the survey in its entirety.

## Data Analysis

A mixed methods approach was used for survey analysis. Descriptive statistics such as frequency analysis were used to examine closed-ended questions, while a qualitative approach was used to identify overarching themes for open-ended questions. Survey results were imported into Microsoft Excel and STATA for analysis.

Four open-ended questions gave volunteers the opportunity to enter free text. One of these questions centered around understanding motivations for volunteering for The Addis Clinic. Specifically, respondents were asked what they enjoyed most about volunteering. Only responses from active volunteers were analyzed. Survey responses were thematically analyzed using the Volunteer Functions Inventory (VFI). This approach examines six motivational functions or motives for volunteering (20). It assumes that individuals volunteer their time and skills for the same organization for different reasons. The six motivational functions include:

1. Values—altruistic motivation and concern for others
2. Understanding—increase knowledge and gain new skills
3. Social—engage with others and/or participate in activities highly regarded by others
4. Career—satisfy career-related objectives and goals
5. Protective—reduce guilt and/or manage inner struggles
6. Enhancement—validate self-worth and/or feel needed by society.

Three members of the research team (EK, SF, MM) reviewed the responses independently. Each reviewer assigned the most appropriate function based on his/her impression of the data. If more than one motivational function applied, reviewers had the option to identify up to three functions by prioritizing the most applicable as primary and least applicable as tertiary. Responses that did not fit any of the functions were identified as “Other.” Results were validated by triangulating the reviewers' assessments and the frequency for each motivational function was tabulated.

Fisher's exact tests or chi-squared tests were used to compare survey responses between active and inactive participants. Two-sided *p*-values of 0.05 or less were deemed to meet statistical significance.

## Results

### Demographic Characteristics

Among 69 active and 25 inactive volunteers, survey response rates of 74 and 72% were attained, respectively. More than 95% of the physician volunteers resided in the United States (U.S.), while the remaining 5% resided in Canada or the United Kingdom. Demographic characteristics for both groups are shown in Table 1.

**Table 1:** Demographic characteristics of physician volunteers.

	**Active**	**Inactive**	***P*-value**
Sex			0.89
N	49	17	
Male	51%	53%	
Female	49%	47%	
Age			0.001
N	51	18	
20–29 years old	0%	22%	
30–39 years old	51%	17%	
40–49 years old	27%	50%	
50–59 years old	14%	11%	
60 years or older	8%	0%	
Marital status			0.61
N	51	18	
Single	18%	11%	
Married	76%	78%	
Committed relationship	4%	11%	
Other (specify)	2%	0%	
Do you have children under the age of 18?			0.56
N	51	18	
Yes	59%	67%	
No	41%	33%	
How important is religion/faith in your life?			0.21
N	50	18	
Extremely important	48%	33%	
Very important	16%	6%	
Moderately important	10%	11%	
Slightly important	10%	33%	
Not at all important	16%	17%	
Work status			0.67
N	51	18	
Full-time	78%	94%	
Part-time	12%	6%	
Retired	2%	0%	
Other	8%	0%	
Practice type			0.18
N	51	18	
Academic institution	35%	50%	
Hospital employee	18%	17%	
Private practice	22%	33%	
Military	6%	0%	
Other	20%	0%	
Years practicing medicine			0.34
N	51	18	
Less than 1 year	2%	6%	
1–4 years	27%	22%	
5–9 years	27%	11%	
10–14 years	16%	28%	
15–19 years	10%	22%	
>20 years	18%	11%	
Salary (dollars per year)			0.56
N	51	18	
Less than $100,000	20%	33%	
$100,000–$199,999	32%	11%	
$200,000–$299,999	14%	11%	
$300,000–$399,999	10%	17%	
$400,000–$499,999	2%	0%	
$500,000 or more	4%	6%	
I prefer not to answer	18%	22%	
Weeks of vacation per year			0.21
N	43	17	
None	0%	6%	
N/A	0%	0%	
1–2 weeks	12%	0%	
3–4 weeks	63%	65%	
5 or more weeks	26%	29%	
Telemedicine experience			0.43
N	51	18	
No	73%	94%	
Yes (domestic)	14%	6%	
Yes (international)	6%	0%	
Yes (domestic and international)	8%	0%	
Time as volunteer [years, median (IQR)]	2 (1–3)	3 (2–6)	0.07

Notably, in this cohort, active, as compared with inactive, volunteers were more likely to be <40 years old (51 vs. 39%, *p* = 0.01). Of trends that did not reach statistical significance, active participants were less likely to have practiced medicine for >10 years (34 vs. 61%), work full-time (78 vs. 94%), and be employed at an academic institution (35 vs. 50%). Additionally, other non-significant trends showed that active volunteers were more likely to view religion/faith as personally important (64 vs. 39%) and earn <$200,000 per year (52 vs. 44%). Volunteer cohorts exhibited comparable distributions across sex, marital status, children, and weeks of vacation per year. More than half of respondents for both active and inactive volunteers had children under the age of 18 (59 vs. 67%), were married (76 vs. 78%), and had 3–4 weeks of vacation per year (63 vs. 65%). Both cohorts were relatively equivalent in terms of gender distribution.

### Volunteer Experience/Background

There was a wide range of medical specialties represented among survey participants. Active volunteers listed more than 20 specialties, however, nearly half specialized in primary care specialties such as Pediatrics (12%), Internal Medicine (20%), and Family Medicine (14%). Inactive volunteers represented 12 specialties (39% of which were in primary care specialties), including Internal Medicine (22%), Pulmonology (17%), and Radiology (17%). Importantly, we found that 46% of active participants (*n* = 51) were in primary care specialties, which was not significantly more than 39% of the inactive (*n* = 18) participants (*p* = 0.65).

A vast majority of volunteers, both active (60%) and inactive (100%), were first introduced to The Addis Clinic through word-of-mouth from friends, current and past volunteers, colleagues, or members of the Board of Directors/staff. Active volunteers were also introduced to The Addis Clinic via internet search (17%), conference proceedings (13%), and social media (10%).

**Figure 1:**
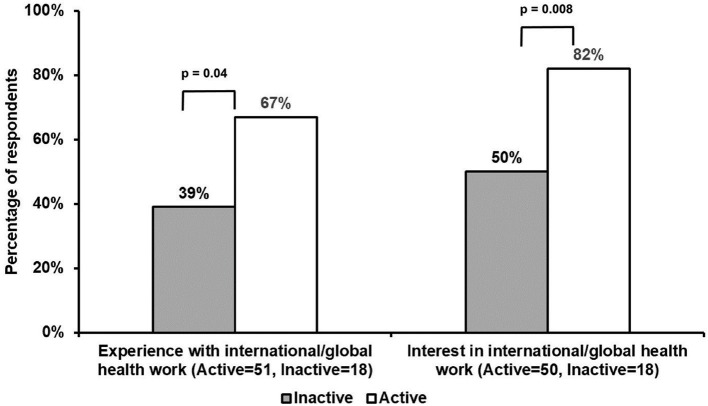
Comparisons of international/global health experience and interest among active and inactive volunteers. Active, as compared with inactive, respondents significantly had greater prior experience with international/global health work (67 vs. 39%, *p* = 0.04). Additionally, active participants were significantly more likely to express an interest in international/global health, as compared with inactive respondents (82 vs. 50%, *p* = 0.008).

Active, as compared with inactive, participants were significantly more likely to have had prior experience with international/global health (67 vs. 39%, *p* = 0.04), as well as express an interest in international/global health (82 vs. 50%, *p* = 0.008), with several active volunteers having prior experience on short-term medical trips (Figure 1). Though not a significant finding, active respondents tended to have greater experience with providing telemedicine services both domestically and/or internationally (28%) (Table 1). The small number of inactive volunteers that reported telemedicine experience had done so in a domestic setting only (6%).

### Barriers to Volunteer Work

**Figure 2:**
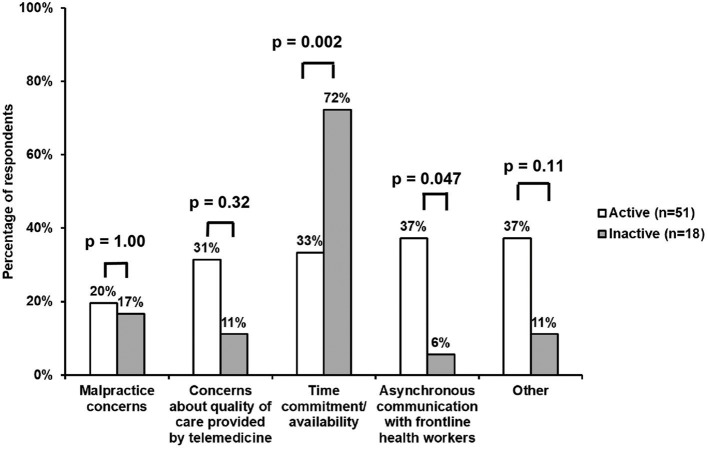
Survey responses regarding barriers to volunteering. Time commitment/availability were more often a significant barrier for inactive, as compared with active, volunteers (72 vs. 33%, *p* = 0.002). On the other hand, the asynchronous communication with FHWs was a significant barrier for a larger percentage of active vs. inactive participants (37 vs. 6%, *p* = 0.047). Furthermore, both active and inactive respondents reported similar results across the other barriers, including concerns about the quality of care provided by telemedicine (31 and 11%, respectively), the asynchronous nature of communication with FHWs (37 and 6%, respectively), and “Other” issues (37 and 11%, respectively). Malpractice concerns were cited in a comparable minority for both active and inactive volunteers (20 and 17%, respectively).

Responses regarding barriers to volunteering are summarized in Figure 2. A comparable minority for both active and inactive volunteers cited malpractice concerns, particularly in the international setting (20 and 17%, respectively). Time commitment/availability were less often a significant barrier for active, as compared with inactive, volunteers (33 vs. 72%, *p* = 0.002). On the other hand, the asynchronous nature of communication with FHWs was reported as a significant barrier for active, as compared with inactive, volunteers (37 vs. 6%, *p* = 0.047). A consistent minority among active volunteers also cited other non-significant barriers, including questions about the quality of care provided by telemedicine (31%). A trend toward a lower percentage of inactive participants voiced the same concerns (11%). Additional concerns (37%) that were not significant, shared mainly among active volunteers (“Other”), included an inability to follow-up with patients and the uncertainties of treating lesser known diseases more commonly seen in LMICs.

**Figure 3:**
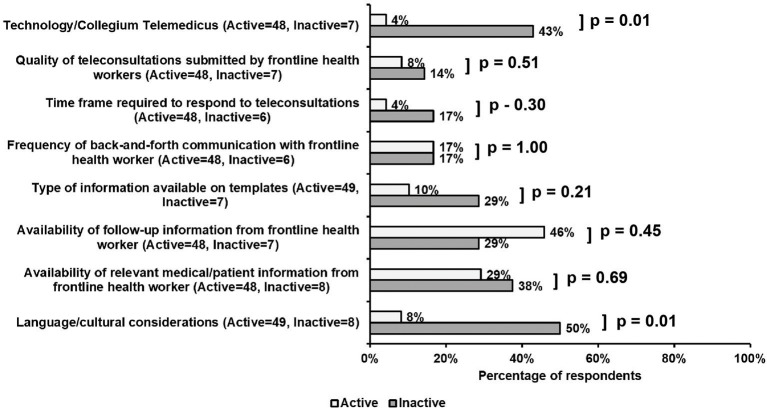
Survey responses regarding perceived obstacles to the teleconsultation process. Noteworthy and significant differences between active and inactive participants included difficulties with language/cultural considerations (8 vs. 50%, *p* = 0.01), and the telemedicine platform interface (4 vs. 43%, *p* = 0.01). Active respondents also found the availability of follow-up (46%) and relevant medical (29%) information from FHWs as obstacles to the teleconsultation process. Features of work-flow were not viewed as an obstacle by a majority of either cohort.

Aspects of the teleconsultation process participants found challenging are summarized in Figure 3. Active, compared with inactive volunteers, were less likely to report language/cultural considerations (8 vs. 50%, *p* = 0.01), and the technology interface (4 vs. 43%, *p* = 0.01) as significant barriers to the teleconsultation process. Though not a significant difference, active volunteers were more concerned with aspects of patient care, with 46% citing the availability of follow-up information from FHWs and 29% questioning the availability of relevant medical/patient information from FHWs. Features of work-flow were not seen as an obstacle by a majority of either active or inactive volunteers. Less than 20% of active and inactive respondents found the frequency of back-and-forth communication with FHWs as problematic, while 4% active and 17% inactive claimed the time frame (24–48 h) required to respond to teleconsultations as difficult.

### Motivations for Volunteer Work

Motivations toward volunteering varied among active volunteers. A frequency distribution table (Table 2) summarizes the results of the VFI analysis.

**Table 2:** Motivational functions for volunteering.

	**Primary**	**Secondary**	**Tertiary**
Values	70	7	0
Understanding	32	11	0
Social	5	1	0
Career	0	2	5
Protective	1	1	0
Enhancement	12	5	2
Other	9	0	0

Notably, common themes emerged throughout the responses. The ability to help patients in resource-constrained environments was most important to active volunteers, while the motivation to better understand a different culture and gain new skills was the second most common motivation:

“*Sense of providing service to a patient that otherwise would not have access to a specialist.”*

“*Helping to provide a service otherwise not available to those who need it.”*

“*Interesting to see the cases detailed in the newsletter and learning how to care for patients in settings with limited resources.”*

In some cases, the reasons for volunteering were more complex with more than one motivational function attributed to a participant's response. Underlying secondary and tertiary themes emerged for a handful of respondents where, for example, both the “values” and “understanding” functions were applicable:

“*Learning about patients who live in the country I once lived and being able to get the chance to contribute.”*

**Figure 4:**
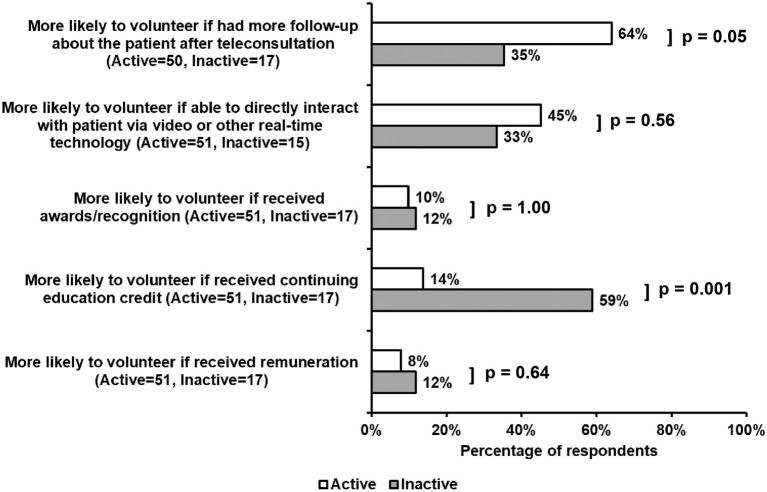
Survey responses regarding incentives for volunteer work. Increased patient follow-up significantly increased enthusiasm among active, as compared with inactive, volunteers (64 vs. 35%, *p* = 0.05). Additionally, the use of real-time technology was viewed as an incentive to volunteering for both active and inactive cohorts (45 and 33%, respectively). Alternatively, the opportunity to receive continuing education credit was a significant incentive for inactive volunteers, as compared with active respondents (59 vs. 14%, *p* = 0.001). Opportunities to receive remuneration or recognition did not increase the likelihood of volunteering for either cohort.

Active and inactive volunteers differed in their views of incentives for volunteering (Figure 4). A higher percentage of active, compared to inactive, volunteers felt that more patient follow-up would significantly increase their enthusiasm for volunteering (64 vs. 35%, *p* = 0.05). Though not a significant finding, the use of real-time technology for direct encounters was viewed as a potential incentive among both active and inactive volunteers (45 vs. 33%). Furthermore, active, as compared with inactive volunteers, were significantly less likely to be incentivized if given opportunities to fulfill professional obligations and receive continuing education credit (14 vs. 59%, *p* = 0.001). Interestingly, opportunities to receive remuneration or recognition were unlikely to incentivize volunteering for either cohort.

A large majority of active volunteers were satisfied with The Addis Clinic teleconsultation process (*p* < 0.0001), with 86% citing satisfaction in general and 78% citing satisfaction with the cases submitted by FHWs (Table 3). Inactive volunteers; however, were indifferent to the teleconsultation process, with 86% claiming neither satisfied nor dissatisfied and 14% reporting dissatisfied. Similarly, 83% of inactive volunteers were neither satisfied nor dissatisfied with the cases submitted by FHWs.

**Table 3:** Satisfaction with the teleconsultation process.

	**Active**	**Inactive**	***P*-value**
N	51	7	<0.0001
Satisfied	86%	0%	
Neither satisfied nor dissatisfied	8%	86%	
Dissatisfied	6%	14%	

## Discussion

### Summary of Findings

By surveying active and inactive physician volunteers at a single humanitarian telemedicine organization (The Addis Clinic), our results reveal several critical factors for motivating and maintaining physician volunteerism. Younger age and having global health experience/interest were significant predictors of consistent physician volunteerism. While inactive volunteers reported time commitment/availability as a significant barrier to volunteer work, active participants were concerned with challenges regarding patient care and information exchange. Additionally, active volunteers were motivated by increased methods to connect with patients. Surprisingly, financial considerations and recognition were infrequently reported as a barrier to volunteer work. Together, these results serve as a valuable guide toward optimizing the strategies for recruiting and maintaining engaged physician volunteers in international telemedicine.

### Barriers

Our data indicated that specific demographic features were associated with a greater willingness to volunteer. These demographics, at least in part, may help to explain the fact that time commitment/availability was ranked more frequently as a predominant barrier to volunteering by inactive respondents. That is, more than half of the inactive volunteers were >40 years old and at the peak of their professional careers. Additionally, 67% of inactive respondents reported having school-aged children. Thus, it is possible that challenges of work-life balance dissuaded inactive participants from committing to volunteering further or at all. Furthermore, it is reasonable to extrapolate that coupling their limited time availability with minimal experience and interest in global health and telemedicine would create challenges in maintaining longitudinal interest in volunteering. Our study also found that language and cultural considerations created a significant barrier for inactive volunteers, similar to reports from other international volunteers participating on short-term assignments (6, 19). These insights suggest that humanitarian telemedicine organizations such as The Addis Clinic could preferentially recruit physicians at specific life stages and fitting certain demographic profiles that are more amenable to volunteerism. Future surveys of a larger cohort of inactive volunteers over a broader time window will also be valuable to ensure consistency of perspectives across multiple cycles of participants.

Active participants; however, described a broader scope of barriers with telemedicine that thematically centered on challenges of patient care and information exchange (Figure 2). Furthermore, over one-third of all active volunteers found the asynchronous communication with FHWs as a significant barrier. This may be linked to the result that nearly half of active respondents were more likely to volunteer if they were able to directly interact with patients via real-time technology. Additionally, greater follow-up information about the patient significantly increased enthusiasm among active volunteers, especially for complex cases. Active participants desired more information on the local resources available to ensure the correct diagnosis and management plan was in place. These principles may also reflect a difference in motivation for physicians volunteering longitudinally as compared with more short-term programs where retention of physician motivation may be less important.

Furthermore, shared themes may underlie the breadth of barriers described by active participants, including notions that physician volunteers from developed countries often are unaccustomed to the pace and workflow processes common in LMICs (6). Physician volunteers may find the store-and-forward nature of asynchronous telemedicine cumbersome because of the inability to receive immediate feedback. Moreover, managing the continuum of care, including a slower process of gathering follow-up information, may be an unfamiliar challenge in resource-rich health systems.

In the future, as telemedicine programs grow in developed countries, it is likely that some of these challenges will become less foreign to physicians who volunteer virtually^3^. Additionally, efforts from The Addis Clinic are underway to initiate a more structured physician education platform unique to practicing telemedicine in resource-poor environments. Such a program could be effective in bridging the cultural and medical disconnect among active volunteers and FHWs working in remote, hard-to-reach areas.

### Motivations

It is notable that the potential for greater patient contact in general increased enthusiasm among active volunteers. This may stem from the fact that contemporary U.S.-trained physicians in general have been vocal about a desire to establish direct patient-physician relationships, despite geographical or other logistical barriers (21). This sentiment may also encapsulate specific root causes of U.S. physician dissatisfaction and burnout, where time to interact with patients continues to diminish in lieu of increased demands for paperwork, and insurance documentation by physicians on electronic health record systems (21). These notions also correspond with the results from the VFI analysis (Table 2) that demonstrated the “values” function as the primary motivation for volunteering among active respondents. Additionally, the desire to help and concern for others could be augmented by the fact that the majority of active volunteers are foreign-born physicians. With the rise in healthcare professionals seeking global health experiences, it may be advantageous for organizations to target foreign-born physicians as volunteers. Certainly, video and real-time technologies as methods for more direct patient contact are already available for international telemedicine endeavors (22). However, real-time video-based appointments across global time zones would require a much more structured and often inflexible demand on physician volunteers' schedules—a notion that is at odds with the barrier of time commitment/availability. Based on these results, innovative ideas to reconcile these seemingly opposing motivations and barriers to volunteering are worth exploring.

Interestingly, a number of inactive participants stated that they were more likely to volunteer if receiving professional incentives such as continuing education credit. Since most of the inactive volunteers were at the peak of their careers and dealing with limited time availability, it could be reasonable to infer that inactive respondents may be more amenable to volunteer if it would also maximize their opportunities to fulfill additional personal and/or career-related obligations. However, our results also revealed that such fulfillment would not necessarily be embodied by personal financial gain. In fact, monetary compensation did not serve as a key motivation for volunteering in a majority of both active and inactive volunteers, consistent with prior researchers finding humanitarian and altruistic rationale as a central driver for international volunteers (23, 24). Thus, guided by these findings, it may behoove The Addis Clinic and other like-minded organizations to partner with physician employers to provide innovative mechanisms facilitating physician promotion and/or offering time-saving measures for physicians in other aspects of the work day, if choosing to volunteer in international telemedicine service. Offering malpractice coverage for international telemedicine may also be effective in mitigating the concerns of the ~20% of participants in both active and inactive cohorts.

### Study Limitations

All participants were volunteers for a single telemedicine organization, which incurs bias and may limit generalizability to other organizations. Additionally, there was a relatively small pool of inactive physician volunteers, as volunteer retention for The Addis Clinic remains high, with fewer than 20% of volunteers leaving the organization since its founding. Furthermore, many of the inactive volunteers have not been recently involved with The Addis Clinic. Consequently, some of their perceived barriers to the teleconsultation process may not be as relevant in the current era of the program, given the significant organizational changes since 2016. For instance, nearly half of the inactive respondents noted the telemedicine technology as a major barrier to volunteering; however, only 4% of active respondents agreed. It is important to note that The Addis Clinic switched to a separate telemedicine platform in 2017. To address this limitation, future studies could be structured to solicit feedback from volunteers prospectively as they leave the organization. Additionally, given the small sample size for both the active and inactive cohorts, more detailed analyses regarding the specific roles of various demographic characteristics on volunteerism were challenging. To better understand whether demographics were factors in the responses of volunteers, larger sample sizes will be needed to maximize statistical power. Furthermore, the VFI analysis found that the motivations for volunteering are complex, with more than one motivational function attributed to a participant's response. The scope of this study provided an initial interpretation of the qualitative data, but additional studies are warranted to expand on the findings. Finally, this study was not designed to compare accuracy and efficacy of physician volunteer diagnoses and recommendations, which could play a central role in reinforcing vs. reducing long-term physician motivations. Given these emerging concepts revealed in this initial study, a more in-depth analysis across multiple telemedicine organizations would be valuable to better understand the underlying thought processes for physicians choosing to volunteer virtually.

## Conclusion

By harnessing the power of telemedicine technology coupled with physician volunteerism, international telemedicine programs have the potential to strengthen the local health workforce and improve health outcomes in remote communities. Guided by the principles revealed in this proof-of-concept study, it will be important for organizations to develop volunteer programs that are mutually beneficial to physician volunteers and partner organizations to ensure long-term interest. More research is needed to better understand the sustainability and potential for large-scale implementation of organizations using virtual volunteers. Organizations may consider applying these findings to develop focused volunteer recruitment and retention strategies.

## Data Availability

The datasets generated for this study are available on request to the corresponding author.

## Author Contributions

EK, SF, MT, TG, and SC conceived the study, designed the survey and analysis, and wrote the manuscript. EK and TG conducted the quantitative analysis. EK, SF, and MM performed the Volunteer Functions Inventory analysis. All authors participated in interpreting the results and revising the manuscript.

### Conflict of Interest Statement

SC currently is the Chair of the Board of Directors of The Addis Clinic, Inc., and holds a faculty position at the University of Pittsburgh School of Medicine and UPMC. SC has served as a consultant for Zogenix, Aerpio, Vivus, and United Therapeutics. SC holds research grants for Pfizer and Actelion. SC holds positions as director, officer, and shareholder of Numa Therapeutics. SC has served as inventor of patent applications regarding new therapies in pulmonary hypertension. The remaining authors declare that the research was conducted in the absence of any commercial or financial relationships that could be construed as a potential conflict of interest.

## Footnotes

^1^https://www.addisclinic.org/

^2^https://www.collegiumtelemedicus.org/ct/index.php

^3^https://www.mordorintelligence.com/industry-reports/global-telemedicine-market-industry
